# The impact of temperature and relative humidity on SARS-CoV-2 airborne transmission in Syrian hamsters

**DOI:** 10.1128/spectrum.00979-25

**Published:** 2025-07-22

**Authors:** Chunmao Zhang, Zhendong Guo

**Affiliations:** 1 Changchun Veterinary Research Institute, Chinese Academy of Agricultural Sciences 595703 , Changchun, China; National Microbiology Laboratory, Winnipeg, Manitoba, Canada

**Keywords:** SARS-CoV-2, airborne transmission, temperature, humidity, Syrian hamster, seasonality

## Abstract

Numerous studies have analyzed the effect of temperature and humidity on COVID-19 transmission, but the findings are inconsistent. This was due to differences in research methodologies, study regions and periods, selected variables, and confounding factors. Using Syrian hamsters, we conducted an evaluation of the airborne transmission of severe acute respiratory syndrome coronavirus 2 (SARS-CoV-2) under various temperature and relative humidity combination conditions in a tightly controlled environmental chamber. We found that the airborne transmission of SARS-CoV-2 was moderately slowed in high temperature and high relative humidity conditions. This can be partially attributed to reduced replication in hamsters and the shortened survival time of the virus in the air. In this study, we have demonstrated that the transmission of SARS-CoV-2 among hamsters is co-modulated to some extent by temperature and relative humidity, and the effect is less pronounced than anticipated that high temperatures would block or substantially reduce SARS-CoV-2 airborne transmission.

**IMPORTANCE:**

In this study, we found that severe acute respiratory syndrome coronavirus 2 (SARS-CoV-2) airborne transmission is to some extent co-modulated by temperature and humidity. However, solely relying on seasonal fluctuations in temperature and humidity is insufficient to substantially mitigate COVID-19 transmission, underscoring the critical need for sustained public health measures. Based on our findings in this hamster model, we infer that COVID-19 may have some seasonal patterns, but these patterns would not be as conspicuous as those of influenza. This insight has significant implications for the ongoing efforts in monitoring, preventing, and controlling the spread of COVID-19.

## INTRODUCTION

At the beginning of the COVID-19 pandemic, the role of environmental factors, particularly temperature and relative humidity, in modulating severe acute respiratory syndrome coronavirus 2 (SARS-CoV-2) transmission has received great attention; however, the topic remains highly controversial. Numerous studies have analyzed the relationship between environmental factors and virus transmission, but the findings are divergent and inconsistent. This may be due to differences in research methodologies, study regions and periods, considered variables, and confounding factors.

Regarding the relationship between temperature and SARS-CoV-2 transmission, some studies reported negative correlations (1), while others have found no association (2) or even positive correlations within specific temperature ranges (3). For instance, a multicity study in China showed that low temperatures and low humidity favored the virus transmission (1), whereas another study in Chinese cities reported no association between COVID-19 transmission and temperature (2). Wagatsuma’s study revealed that extremely hot and humid weather only marginally reduced time-varying effective reproduction number and COVID-19 transmission in Japan (4). Surprisingly, another study found a positive linear association between mean temperature and COVID-19 cases with a threshold of 3℃ and further noted that there was no evidence to support the hypothesis that COVID-19 cases would decline when the weather becomes warmer (3).

Similarly, studies on the relationship between humidity and SARS-CoV-2 transmission have yielded conflicting results, ranging from negative to positive correlations (5–7). For instance, one study showed that both humidity and temperature were negatively related to the daily new cases and daily new deaths of COVID-19 in 166 countries (5), whereas another study exploring COVID-19 transmission in Africa showed that the relative humidity and daily new cases of COVID-19 were positively correlated in Tunisia, but were negatively associated in South Africa, Ethiopia, and Morocco (6). Furthermore, a study conducted in the mainland US reported that the relationship between relative humidity and COVID-19 spread could be either positive or negative, depending on the specific regions and conditions (7). Guise Erlenmeyer et al. reported that there was no significant association between relative humidity and COVID-19 incidence in their study (8).

The laboratory studies, conducted in tightly controlled conditions with the selected variables, can avoid the adverse influence of confounding factors and overcome the shortcomings of epidemiological studies. Syrian hamsters, as a small animal model, have been widely used to study the pathogenesis and transmission of SARS-CoV-2 (9, 10). Accumulating evidence suggests that the primary mode of SARS-CoV-2 transmission is airborne transmission from close contact with the infected COVID-19 patients (11, 12). To assess the effect of temperature and humidity on SARS-CoV-2 airborne transmission, we conducted an evaluation of SARS-CoV-2 airborne transmission among hamsters under different temperature and relative humidity combination conditions in an environmental chamber. Our findings showed that very high temperature and high humidity moderately reduced SARS-CoV-2 airborne transmission. This study enhanced our understanding of how climate factors may influence the COVID-19 spread in real-world settings.

## RESULTS

### Very high temperature and high humidity slowed SARS-CoV-2 airborne transmission

**Fig 1:**
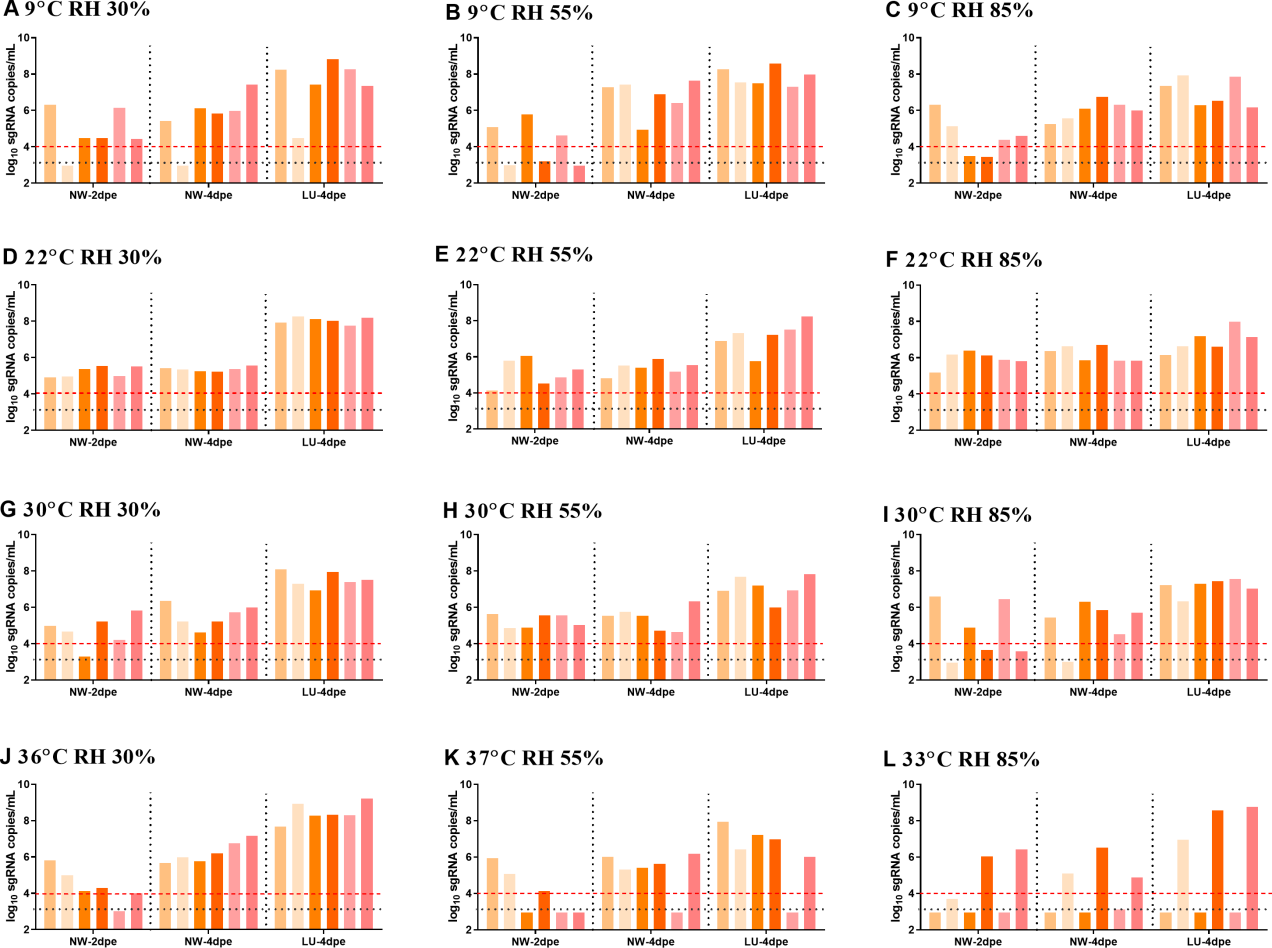
SARS-CoV-2 transmission under different temperature and relative humidity combination conditions. Viral sgRNA load in nasal washes (NW) of the recipient hamsters at days 2 and 4 post-exposure, and in lungs (LU) at day 4 post-exposure in different conditions tested. (A) 9℃ and 30% RH, (B) 9℃ and 55% RH, (C) 9℃ and 85% RH, (D) 22℃ and 30% RH, (E) 22℃ and 55% RH, (F) 22℃ and 85% RH, (G) 30℃ and 30% RH, (H) 30℃ and 55% RH, (I 30℃ and 85% RH, (J) 36℃ and 30% RH, (K) 37℃ and 55% RH, (L) 33℃ and 85% RH. The black dashed line represented the limit of detection, and bars with the same color represented data from the same hamster. The red dashed line represented the cutoff line for positive infection discrimination of the hamsters in the transmission groups. The limit of detection of sgRNA is 1000 sgRNA copies/mL or 3 log sgRNA copies/mL. The cutoff value of sgRNA load for positive infection discrimination of the recipient hamsters is greater than or equal to 10^4^ copies/mL or 4 log sgRNA copies/mL.

To evaluate the impact of temperature and relative humidity on SARS-CoV-2 airborne transmission, we conducted the transmission experiments under various combinations of temperature and relative humidity levels. At low temperature, transmission efficiencies fluctuated moderately, ranging from 5/6 to 3/6 and 4/6 at day 2 with the humidity levels varying from low to high, but achieving full transmission (6/6) at day 4 across all humidity levels (Fig. 1A through C).

At room temperature, SARS-CoV-2 transmitted efficiently between hamsters across all humidity levels, with a transmission efficiency of 6/6 at days 2 and 4 (Fig. 1D through F). At high temperature and low humidity, the transmission efficiency was 5/6 at day 2 but full transmission (6/6) at day 4 (Fig. 1G), and at moderate humidity, the transmission efficiency was 6/6 at days 2 and 4 (Fig. 1H). However, at high humidity, transmission was slowed, with only 3/6 positive nasal washes at day 2 and 5/6 at day 4, and all recipient hamster lungs tested positive at day 4 (Fig. 1).

At 36℃ and 30% RH, the transmission efficiency was 5/6 at day 2 and 6/6 at day 4 (Fig. 1J). At 37℃ and 55% RH, transmission was slowed to 3/6 at day 2 and 5/6 at day 4 (Fig. 1K). Due to Syrian hamsters’ intolerance to the extremely high temperature and high humidity conditions (37℃, 85% RH), the experimental temperature was reduced to 33–34℃. At 33℃ and 85% RH, transmission was slowed, with efficiencies of 2/6 at day 2 and 3/6 at day 4 (Fig. 1L). At high RH, increasing temperature gradually slowed SARS-CoV-2 transmission. At 30℃ or higher, increasing humidity also led to a gradual decrease in transmission. Overall, the high temperature and high humidity combination was unfavorable for SARS-CoV-2 airborne transmission.

### High temperature slowed SARS-CoV-2 transmission in a short-term exposure

In a real-life scenario, SARS-CoV-2 transmission often occurs within a short time frame. The results of our study have shown that high relative humidity only reduces SARS-CoV-2 transmission at very high temperatures, whereas very high temperatures and high temperatures can moderately or slightly reduce SARS-CoV-2 transmission at more humidity levels. To some extent, it seems that the temperature on SARS-CoV-2 transmission may impact more widely than humidity. Furthermore, considering the experiences of the epidemic of seasonal coronaviruses and influenza viruses, high temperature may be more closely associated with the seasonality of these viruses. Hence, we focused on temperatures and only tested the effect of high temperatures on transmission efficiency during short-term exposure. To mimic this condition, we conducted transmission experiments with shortened exposure duration of 1 h and 3 h and evaluated SARS-CoV-2 airborne transmission at 22℃ and 37℃.

**Fig 2:**
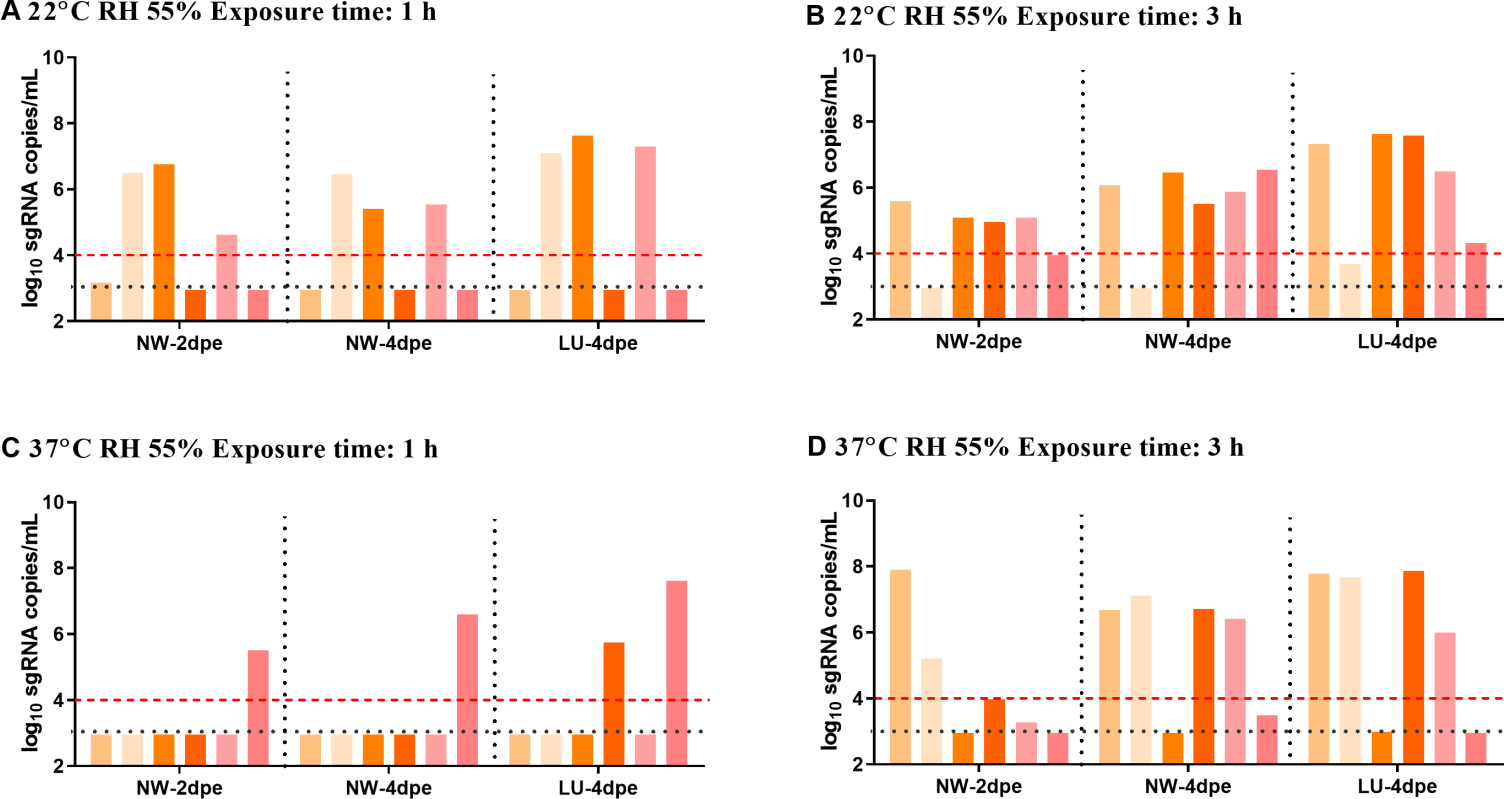
SARS-CoV-2 transmission in short-term exposure. Viral sgRNA load in nasal washes (NW) at days 2 and 4 post-exposure, and in lungs (LU) at day 4 post-exposure in different combinations of temperature and exposure time. (A) 22℃ and 1 hour, (B) 22℃ and 3 hours, (C) 37℃ and 1 hour, (D) 37℃ and 3 hours.The black dashed line represented the limit of detection, and bars with the same color represented data from the same hamster. The red dashed line represented the cutoff line for positive infection discrimination of the hamsters in the transmission groups.

At 22℃ with a 1 h exposure, the transmission efficiency was 3/6 at days 2 and 4 (Fig. 2A). Remarkably, at 37℃ with a 1 h exposure, the transmission efficiency was slowed to 1/6 at day 2, and 1/6 and 2/6 in nasal washes and lungs at day 4 (Fig. 2C). At 22℃ with a 3 h exposure, the transmission efficiency was 4/6 at day 2 and 5/6 at day 4 (Fig. 2B). However, at 37℃ with a 3 h exposure, transmission efficiency was slowed to 2/6 at day 2 and 4/6 in both nasal washes and lungs at day 4 (Fig. 2D). These findings indicate that high temperature can mitigate the short-term airborne transmission of SARS-CoV-2.

### The very high temperature reduced SARS-CoV-2 replication in hamsters

**Fig 3:**
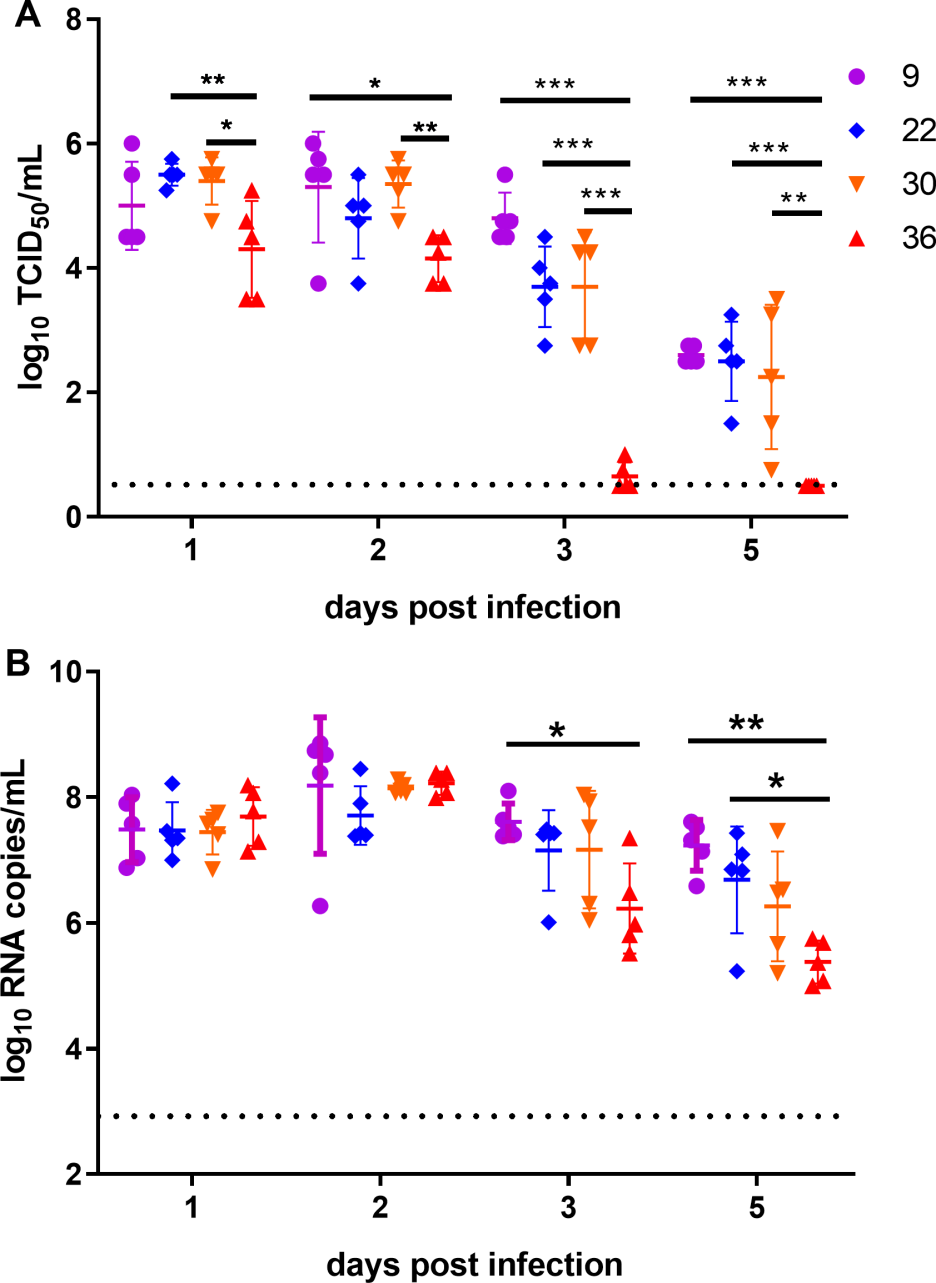
Viral replication in hamster under different temperature levels. (**A**) Viral titers (mean ± sd) and (**B**) viral RNA load (mean ± sd) in nasal washes (NW) of hamster at post-infection days. One-way analysis of variance (ANOVA) test was used to analyze the significant differences in viral titers and viral RNA in NW between different groups (*P < 0.05; **P < 0.01; ***P < 0.001). The dashed line represented the limit of detection. The limit of detection for viral titer was 0.5 log_10_TCID50/mL, and the limit of detection for genomic RNA load was 1000 copies/mL or 3 log_10_ RNA/mL.

Efficient replication and release of the virus are crucial for its airborne transmission. In this study, we determined the replication dynamics of SARS-CoV-2 in the upper respiratory tract of hamsters across various temperature levels. On day 1, viral titer in nasal washes from hamsters at 9℃ displayed moderate variations but generally higher than that at 36℃, although the difference was not statistically significant (Fig. 3A). The viral titers at 22℃ and 30℃ were significantly higher than that at 36℃ (Fig. 3A). On day 2, the viral titer in nasal washes from hamsters at 9℃ and 30℃ was significantly higher than that at 36℃ (Fig. 3A). The viral titer at 22℃ was numerically higher than that at 36℃, but without statistical significance (Fig. 3A). On day 3, the viral titer in nasal washes from hamsters at 9℃ was higher than that at the other three temperature levels (Fig. 3A). Notably, viral RNA load in nasal washes remained similar at all temperature levels and maintained high levels at days 1 and 2 (Fig. 3B). These findings indicated that very high temperature effectively reduced SARS-CoV-2 replication in the respiratory tracts of hamsters. Furthermore, low temperature moderately extends the replication and shedding period of the virus in hamsters.

## DISCUSSIONS

In this study, we investigate the impact of ambient relative humidity and temperature on SARS-CoV-2 airborne transmission using a hamster model. We test four different temperatures (9–37℃) and three relative humidity conditions (30–85% RH) and find that airborne transmission is surprisingly robust across these ranges. However, transmission efficiency decreased with shorter exposure time at high temperature.

The airborne transmission of SARS-CoV-2 between hamsters was efficient at 30℃, with a transmission efficiency of 100%. Even at 37℃, transmission efficiency was only slightly reduced under moderate humidity. The robust transmission ability at high temperatures contrasts sharply with influenza viruses, with airborne transmission between guinea pigs being blocked at 30℃ (13, 14). SARS-CoV-2 airborne transmission is more resistant to high temperature than influenza viruses. This can be partially attributed to the higher stability of SARS-CoV-2 in the air, with a half-life of 3 h (15), significantly longer than the less than 30 min half-life of influenza A viruses (16).

Three mechanisms could explain how temperature and relative humidity affect SARS-CoV-2 transmission. First, ambient temperatures influence host metabolism and immunity by modulating body temperature. Prior research has shown that higher body temperature increases host resistance to viruses like SARS-CoV-2 and influenza by increasing bile acids production in a gut macrobiotic-dependent manner (17). Our study revealed that SARS-CoV-2 replication decreased significantly in hamsters at 36℃, partially explaining the reduced transmission at very high temperature. Second, many respiratory viruses exhibit thermal sensitivity (18), with their polymer activity and viral replication capabilities being particularly sensitive to variations in temperature. Previous research has shown that SARS-CoV-2 is thermally sensitive (19), and an increase in temperature to 40℃ inhibits SARS-CoV-2 replication and transcription in respiratory epithelial cells (20). In our study, at an ambient temperature of 36℃, the body temperature of hamsters rose to 38–39℃, which may have interfered with the virus’ transcription and replication process, thus reducing its transmission efficiency. Third, the stability of SARS-CoV-2 particles in the air is co-modulated by both temperature and relative humidity. Previous research demonstrated that high temperatures and high humidity are detrimental to the virus’ survival in the air (21), resulting in a decrease in the number of viable virus particles available for transmission and subsequently reducing airborne transmission. Our findings supported this mechanism, as we observed a decrease in SARS-CoV-2 transmission efficiency at high temperature and high humidity (33℃, 85% RH) (Fig. 1L). To be worthy of mentioning, high temperature and high humidity may alter the hamsters’ behavior and activity intensity, and further indirectly impact or reduce viral transmission between hamsters. The reduced transmission may be a combined effect of high temperature and high humidity on SARS-CoV-2 admissibility and on the hamsters themselves. Lastly, relative humidity affects the size and behavior of droplets carrying the virus. At high relative humidity, exhaled droplets evaporate slowly and fall faster, while at low relative humidity, large droplets rapidly shrink and stay airborne longer (22). However, our study found no significant change in transmission efficiencies with increasing humidity at low and room temperature, suggesting that this mechanism may not be a major determinant in SARS-CoV-2 transmission. Our findings suggest that the primary mechanisms by which temperature and humidity affect SARS-CoV-2 transmission include modulating host immunity and metabolism, altering the biological characteristics of the virus itself, and influencing the virus particle survival in the air.

The classic theory says that low temperature and low humidity favor the respiratory viruses’ transmission (23). However, our experiments revealed a moderately slowed SARS-CoV-2 transmission at low temperature compared to room temperature at day 2 post-exposure. Upon closer examination, we observed that viral titer in nasal washes of the infected hamsters at low temperature was slightly lower than that at room temperature at day 1 post-infection, although the difference was not statistically significant (Fig. 3A). This suggests that SARS-CoV-2 initially replicates in hamsters at a slower pace at low temperature. The body temperature of hamsters shows a quick drop when in cold (24), and the respiratory tract temperature decreases further. This may reduce the metabolic activity of cells lining the respiratory tracts and viral replication and release, potentially slowing transmission.

In contrast to influenza viruses, SARS-CoV-2 demonstrates an effective transmission across a wider temperature range. It can effectively transmit through the air between hamsters at ambient temperatures above 30℃. Only high temperature and high humidity combination can moderately reduce its airborne transmission. This suggests that the potential persistence of COVID-19 during summer, albeit with a slight to moderate reduction in epidemic intensity, yet remaining higher than that of influenza. At low and room temperature, SARS-CoV-2 transmission is less sensitive to relative humidity when compared to influenza viruses. The increase in transmission ability in winter may not be as pronounced as that of influenza viruses. To some extent, enviromental factors, such as temperature and humidity, may influence the seasonal patterns of COVID-19, but this seasonality may not be as significant as observed for influenza. It should be noted that humans and hamsters diverge in several aspects. Our experimental findings in the hamster model may not be fully transferable to humans. Special caution is required when extrapolating these findings to the human population. Furthermore, our study did not include other SARS-CoV-2 variants, and several studies have shown that later SARS-CoV-2 lineages vary in environmental stability, replication, and shedding (25–27). We are not sure whether our findings will be applicable to currently circulating viruses. Further study is needed to evaluate the impact of temperatures and relative humidity on the airborne transmission of emerging SARS-CoV-2 variants.

### Conclusion

In this study, we examined how temperature and humidity affect the airborne transmission of SARS-CoV-2 among hamsters. Our results show that high temperature and high humidity together moderately reduce the transmission efficiency, but the effect is less significant than expected. Our findings indicate that solely relying on seasonal fluctuations in temperature and humidity is insufficient to substantially mitigate COVID-19 transmission, underscoring the critical need for sustained public health measures.

## MATERIALS AND METHODS

### Cells and virus

Vero-E6 cells (CRL1586, ATCC, USA) were grown in high-glucose Culbertson’s modified Eagle’s medium (DMEM; Sigma Baldric, USA), supplemented with 10% fetal bovine serum (FBS; Sigma Baldric, USA), 100 U/ml penicillin, and 100 Ag/ml streptomycin, at 37 ℃ with 5% CO2. The SARS-CoV-2 virus, Betake/Beijing/IME-BJ05-2020, was propagated in Vero-E6 cells and arbitrated using the classic 50% endpoint assay based on virus-induced pathogenic effect in Vero-E6 cells.

### Animals, Ethics, and Biosafety

Male Syrian hamsters, 10 to 14 weeks old, were purchased from Charles River Laboratories. All hamsters were housed in individually ventilated cages, allowed access to food and water *ad libitum*, and kept on a 12 h light/dark cycle. This study was conducted in strict accordance with the Guide for the Care and Use of Laboratory Animals of the Ministry of Science and Technology of China. All animal experimental procedures were approved by the Animal Care and Use Committee of Changchun Veterinary Research Institute (the approval number for hamsters is IACUC of AMMS-2020-012). Experiments involving the infectious SARS-CoV-2 virus were performed in the Animal Biosafety Level 3 (ABSL3) laboratory and approved by the Biosafety Committee of the Changchun Veterinary Research Institute. Considering the airborne transmission of SARS-CoV-2, experimenters involving the handling of SARS-CoV-2 infected hamsters in ABSL3 were equipped with positive pressure protective head cover to avoid potential inhalation of viral aerosol.

### SARS-CoV-2 transmission studies in Syrian hamsters

**Fig 4:**
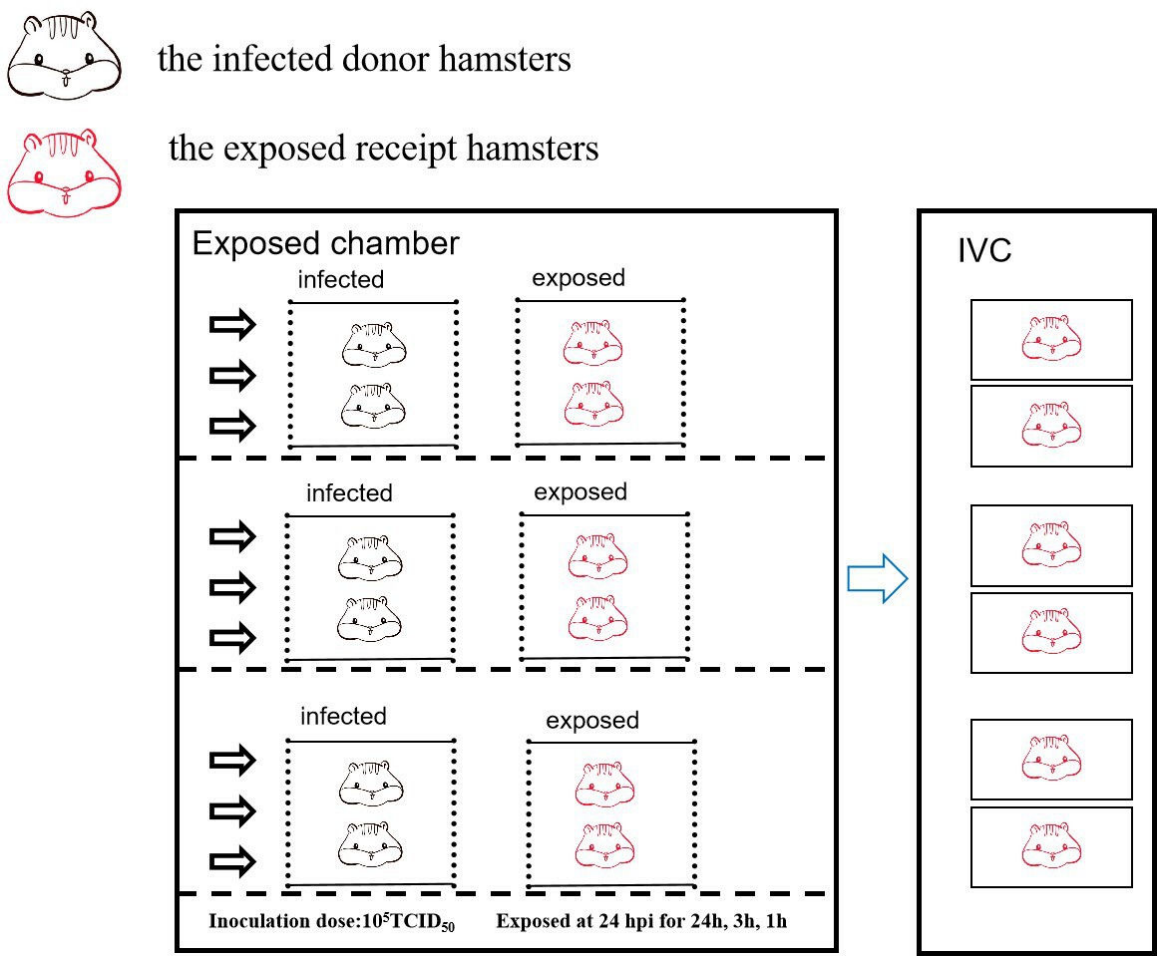
The diagram of the experimental setup for SARS-CoV-2 transmission. In each experiment, 12 hamsters were placed in the environmental chamber. Two hamsters were housed in one cage, and two paired cages were placed on a shelf. The six inoculated hamsters were placed in the three cages on the left, and the six recipient hamsters were placed in the three cages on the right. The air flowed freely from the left to the right. Once the exposure ended, each recipient hamster was transferred to an individually ventilated cage (IVC).

The transmission experiments were conducted in an environmental chamber (LHH250SD, Envying, China) with specific temperature and relative humidity. The arrangement of the cages and hamsters in the chamber is illustrated in Fig. 4. Animals in the chamber were kept on a 12 h light/dark cycle and allowed free access to food and water. The intensity of the light in the chamber in the daytime was 20 lux. The temperature fluctuation range was ±0.5°C, and the relative humidity fluctuation range was ±3% RH. Each transmission experiment involved 12 male Syrian hamsters. On day 0, three groups of two hamsters were anesthetized and intranasally inoculated with 10^5^ TCID_50_ of the virus. Subsequently, they were housed in an environmental chamber at specified environmental conditions. One day post-infection, three additional groups of two naive hamsters were paired with the three previously inoculated groups and transferred to three sets of transmission cages placed in the chamber. The virus-inoculated donor hamsters were placed into cages on the left side, and the naive recipient hamsters were placed into cages on the right. The distance between the two paired cages was about 3 to 4 cm. Both sides of the cages were perforated to allow free airflow from left to right .

After 24 h exposure, each recipient hamster was transferred to an individual ventilated cage and housed for an additional three days at room temperature. Nasal washes were collected from all recipient hamsters using 1 mL of PBS at days 2 and 4 post-exposure. At day 4 post-exposure, all recipient hamsters were euthanized, and their lungs were collected and homogenized in 1 mL of PBS. Viral subgenomic RNA (sgRNA) in nasal washes and lungs was quantified using RT-qPCR. A viral sgRNA load higher than 10^4^ copies/ml in either nasal washes or lungs was considered positive for infection.

A total of 12 experiments were conducted to assess the impact of temperature and relative humidity on SARS-CoV-2 transmission, each involving different combinations of temperature and relative humidity. Temperature was maintained at four levels: low temperature (9℃), room temperature (22℃), high temperature (30℃), and very high temperature (36–37℃). Relative humidity was maintained at three levels: low humidity (30% RH), moderate humidity (55% RH), and high humidity (85% RH). Furthermore, we reduced the exposure time to 1 h or 3 h and repeated the transmission experiments according to the established protocols.

### SARS-CoV-2 replication in Syrian hamsters

To assess the impact of temperature on SARS-CoV-2 replication in hamsters, four groups of five male hamsters were inoculated with 10^5^ TCID_50_ of the virus. Following inoculation, the hamsters were housed in an environmental chamber for 5 days, with each group being maintained at a distinct temperature: 9℃, 22℃, 30℃, and 36℃, respectively. The relative humidity within the chamber was 55%. Nasal washes were collected from all hamsters at days 1, 2, 3, and 5 post-infection. Viral titers in nasal washes were titrated using Vero-E6 cells, and viral RNA load in nasal washes was quantified using RT-qPCR.

### Viral RNA and sgRNA quantification

One hundred microliters of the supernatants of nasal washes and homogenized lungs were used to extract RNA using the Simply P total RNA Extraction Kits (BioFlux, Hangzhou) following the manufacturers’ instructions. We quantified genomic RNA (gRNA) and sgRNA using RT-qPCR as described in previous studies (28, 29). A Ct value <38 is considered positive for both gRNA and sgRNA.

### Statistical analysis

The one-way analysis of variance (ANOVA) test and Tukey’s post-hoc test were used to analyze the statistical differences in viral titers and viral RNA in nasal washes between different groups. When *P* < 0.05, the difference was considered statistically significant. All data were analyzed using GraphPad Prism 6.
